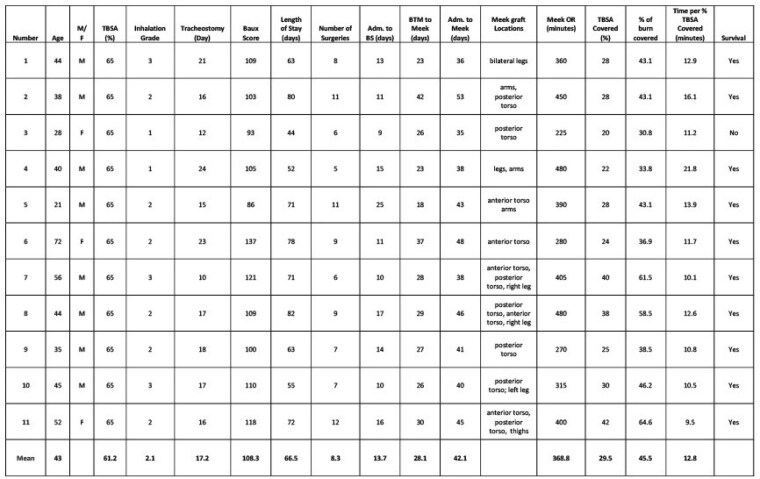# 545 Meek Micrografting and a Bioabsorbable Scaffold for Skin Coverage in Extensive Burn Injuries

**DOI:** 10.1093/jbcr/iraf019.174

**Published:** 2025-04-01

**Authors:** Jacob Wise, David Wallace, Stephanie Mason, Alan Rogers

**Affiliations:** University of Ottawa; Ross Tilley Burn Centre, Sunnybrook Health Sciences Centre; Ross Tilley Burn Centre, Sunnybrook Health Sciences Centre; Ross Tilley Burn Centre, Sunnybrook Health Sciences Centre

## Abstract

**Introduction:**

The management of burn injuries over 50% of the body surface area requires a reliable and reproducible strategy to enhance healing and obtain satisfactory outcomes. This paper explores the application of Meek Micrografting and a bioabsorbable scaffold as a comprehensive treatment approach for these patients at an American Burn Association verified Burn center.

**Methods:**

All patients admitted with full thickness flame burn injuries over 50% TBSA who underwent application of the bioabsorbable scaffold (BS) and Meek micrografting over a two- year period, were included in the study. Data collected included patient demographics, co-morbidities, mechanism and extent of burn injury, surgical approach and hospital course, as well as complications.

**Results:**

Eleven adult patients were included in this retrospective study (Table). Their mean age was 43.2 years (range 21-72 years) and all but three were male. Their mean TBSA burn was 61.2% (range 50 -74%), with Inhalation injury grade of 2.1 and Baux score of 108.3 (range 86-137). Mean length of stay was 66.5 days (range 44-80), and 8.3 surgeries (range 6-12) were undertaken. Tracheostomy was performed at a mean of 17.2 days.

The Meek surgery was usually the final surgery and was performed at a mean of 42.1 days (range 35 – 53 days) after admission. The BS and Meek strategy covered almost half of the burn surface area (mean 29.5% TBSA, or 45.5% of the burn). The 3:1 expansion ratio was used in all patients. The average operative time used per % TBSA covered decreased over the period, with 15.18 minutes per % TBSA during the initial five cases, and 10.8 minutes during the second six cases (p = 0.03). One patient required partial removal of the BS for MRSA infection. Micrograft take exceeded 90% in all cases, and no further autografting was undertaken. All but one of the patients survived to discharge after a period of inpatient rehabilitation.

**Conclusions:**

The bioabsorbable scaffold (BS) provides Meek micrografts with a stable, homogenous, well vascularized and therefore receptive wound bed. We present a series of eleven patients with extensive burns who received the combination of these two strategies to good effect.

**Applicability of Research to Practice:**

With greater experience, burn operating teams can reduce operative time per TBSA covered and obtain excellent outcomes using 3:1 Meek on the wound bed provided by the boabsorbable scaffold.

**Funding for the Study:**

N/A